# Coexistent anemia modulates systemic inflammation and exacerbates disease severity and adverse treatment outcomes in tuberculosis

**DOI:** 10.3389/ftubr.2024.1462654

**Published:** 2025-01-14

**Authors:** Bindu Dasan, Saravanan Munisankar, Nathella Pavan Kumar, Kadar Moideen, Arul Nancy Pandiarajan, Sujatha Nott, Vijay Viswanathan, Sivakumar Shanmugam, Syed Hissar, Kannan Thiruvengadam, Hardy Kornfeld, Subash Babu

**Affiliations:** ^1^Department of ICER, National Institutes of Health-National Institute of Allergy and Infectious Diseases-International Center for Excellence in Research, Chennai, India; ^2^Department of Immunology, Indian Council of Medical Research (ICMR)-National Institute for Research in Tuberculosis, Chennai, India; ^3^Infectious Diseases, Dignity Health, Chandler, AZ, United States; ^4^Prof. M. Viswanathan Diabetes Research Center, Chennai, India; ^5^Department of Bacteriology, ICMR-National Institute for Research in Tuberculosis, Chennai, India; ^6^Department of Clinical Research, ICMR-National Institute for Research in Tuberculosis, Chennai, India; ^7^ICMR-Regional Medical Research Centre, Port Blair, India; ^8^Medicine, UMass Chan Medical School, Worcester, MA, United States; ^9^Laboratory of Parasitic Diseases, National Institutes of Allergy and Infectious Diseases, National Institutes of Health, Bethesda, MD, United States

**Keywords:** anemia, tuberculosis, cytokines, disease severity, treatment outcomes

## Abstract

**Introduction:**

Anemia has been shown to be an independent predictor of disease progression and death in tuberculosis (TB) patients, significantly impacting TB in several ways. This dual burden poses significant challenges for TB control efforts. However, the mechanism by which anemia influences disease severity, bacterial burden, and TB treatment outcomes remains poorly understood.

**Methods:**

In this study, we aimed to compare bacterial burdens, disease severity, and TB treatment outcomes in TB patients with or without anemia. Participants were recruited from Chennai, South India, as part of the prospective Effect of Diabetes on Tuberculosis Severity (EDOTS) study conducted from February 2014 to August 2018. Anemia was defined as hemoglobin (Hb) levels <13 g/dL and <12 g/dL for males and females, respectively. We employed chest X-rays to assess bilateral lung and cavitary diseases and sputum smear grades to measure bacterial loads in TB subjects. Treatment outcomes were defined as favorable or unfavorable. Cytokine profile was measured using multiplex ELISA.

**Results:**

The study comprised of 483 culture-confirmed TB individuals, with 288 positives for anemia {Median Hb was 11.0 [interquartile range (IQR)], 10.3–12.3} and 195 negatives [Median Hb was 14.3 (IQR), 13.5–15.2]. The study revealed that TB patients with anemia had significantly higher bacterial loads [adjusted prevalence ratio (aPR), 4.01; 95% CI, 2.22–6.63; *p* < 0.001], cavitary lung lesions [aPR, 3.36; 95% CI, 1.95–5.68; *p* < 0.001] and unfavorable treatment outcomes [aPR, 1.61; 95% CI, 1.31–2.19; *p* = 0.046] compared to those without anemia. Our data also show that TB is associated with significantly lower levels of type-1 cytokines (IFNγ and IL-2) but significantly higher levels of pro-inflammatory cytokines (IL-6, IFNα, and IFNβ) and pro-fibrotic factors (VEGF, EGF, FGF-2, and PDGF-AB/BB) in anemic individuals compared to those without anemia.

**Conclusions:**

These findings highlight a clear association between anemia and increased TB severity, elevated bacterial loads, and poor treatment outcomes. Our data also suggest that anemia might be associated with the modulation of cytokine responses, which could impart a detrimental effect on TB pathogenesis.

## Introduction

Tuberculosis (TB) remains a major global health threat, resulting in millions of new cases and fatalities annually (1). Anemia is a common risk factor and hematological abnormality associated with TB, with a prevalence of 20–94% in TB patients (2, 3). On the contrary, the likelihood of TB among anemic patients is higher than non-anemic patients (2–4). Anemia is defined as the insufficiency of erythrocyte mass to deliver adequate oxygen to peripheral tissues (5). TB is known to cause “anemia of inflammation” a condition in which systemic inflammation may change iron metabolism and lower red blood cell counts (6). The reduction of erythropoiesis by inflammatory indicators, malabsorption syndrome, and nutritional inadequacies are elucidated as the underlying pathophysiology of anemia in TB patients (7).

Anemia profoundly impacts the course and severity of TB in several ways and has been found to be an independent predictor of disease progression and fatality in TB patients (8, 9). TB patients with anemia have heavy sputum bacillary load and worsened pulmonary infection (10, 11). Studies indicate that anemia is associated with more severe forms of TB and unfavorable disease outcomes, including increased mortality rates and extended treatment periods (12–17). Anemia may increase the risk of complications like pulmonary dysfunction due to larger infectious zones in the lungs, further aggravating TB outcomes (18, 19). Additionally, anemia exacerbates the adverse impacts of TB medications, including gastrointestinal disorders and hepatotoxicity (20, 21).

Cytokines from the innate and adaptive immune systems play crucial roles in orchestrating the immune response to TB. Immune alterations favor the survival, multiplication, and dissemination of *Mycobacterium tuberculosis* (Mtb) and associated sequelae (22, 23). Cell-mediated Th1 immunity, coordinated by Interferon (IFN)-γ, is required to suppress Mtb inside macrophages at the infection site in the lung (24, 25). Th1 cytokines typically activate macrophages and cytolytic T cells to kill intracellular Mtb via the induction of reactive oxygen and nitrogen species, antimicrobial peptides, and autophagy (26). Conversely, Th2 cytokines, such as Interleukin (IL)-4 and IL-13, induce anti-inflammatory reactions that impede pathological inflammation while concurrently impeding macrophage and T cell capacity to efficiently eliminate Mtb (27). Pro-inflammatory cytokines such as IL-6 are multifunctional cytokines that play a crucial role in regulating the immune response, inflammation, and hematopoiesis and are key mediators of anemia of inflammation (24, 28). However, expression of these cytokines in immune responses to TB in anemic individuals have not been explored in detail, and clear data on their impact on bacterial burdens, disease severity, and treatment outcomes are lacking.

To address this knowledge gap, our study aimed to compare bacterial burdens, disease severity, and TB treatment outcomes in TB patients with or without anemia. Moreover, to explore the immunological underpinnings of the interaction between anemia and TB, we examined circulating plasma levels of a large panel of cytokines and pro-fibrotic factors in TB patients with or without anemia.

## Materials and methods

### Ethics statement

The study was approved by the ethics committees of the National Institute for Research in Tuberculosis (NIRT) and the Prof. M. Viswanathan Diabetes Research Center (MVDRC; ECR/51/INST/TN/2013/MVDRC/01).

### Patient consent statement

Informed written consent was obtained from all participants, and study procedures adhered to institutional ethical guidelines.

### Study population and data variables

Participants were recruited from Chennai, South India, as part of the prospective Effect of Diabetes on Tuberculosis Severity (EDOTS) study conducted from February 2014 to August 2018. Anemia was diagnosed based on WHO criteria (hemoglobin concentration < 12 g/dL in women and < 13 g/dL in men) (29). The study included adult individuals aged 25–73 who were newly diagnosed with positive sputum smears and culture. All the participants were screened for diabetes and nutritional indices. Smoking and alcohol consumption status were recorded. Exclusion criteria were previous TB episodes, prior TB treatment, drug-resistant TB, positive HIV status, use of immunosuppressive medications, pregnancy, and lactation. A complete blood count was done on all samples in a DxH 520 hematology analyzer (Beckman Coulter). Anthropometric measurements (height, and weight), and biochemical parameters were procured using standardized techniques. Low body mass index (LBMI) was described based on the American Heart Association/American College of Cardiology guidelines (LBMI ≤ 18.5 kg/m^2^), overweight by body mass index (BMI) 25–29.9 kg/m^2^, and obesity defined by BMI threshold of ≥30.0 kg/m^2^. Diabetes was defined as an glycated hemoglobin (HbA1c) reading of 6.5% or greater and a fasting blood glucose of ≥126 mg/dl, according to the American Diabetes Association criteria. A sample of the individuals with a result of total cholesterol (TC) < 130 mg/dl, triglyceride (TG) < 90 mg/dl, low-density lipoprotein cholesterol (LDL-C) < 100 mg/dl, and high-density lipoprotein cholesterol (HDL-C) < 40 mg/dl were considered as hypolipidemic while individuals with the result of TC ≥ 200, TG ≥ 150, LDL-C ≥ 130 mg/dl, or HDL-C > 40 mg/dl were classified as hyperlipidemic. Vitamin D deficiency was defined as < 30 ng/mL. High or low serum albumin were determined according to serum albumin level of ≥ or < 3.9 g/dl. Chest X-rays were utilized to assess the presence of bilateral lung disease and cavitary lesions and chest x-rays were read by two independent radiologists. Sputum smear grades were used to measure bacterial loads in individuals with TB and classified as 0, 1+, 2+, and 3+ with 0 being no bacteria in microscopy and 3+ the highest number of bacteria. The laboratory investigators were blinded to the chest x-ray and bacteriology results. All recruited TB patients received anti-TB treatment through Directly Observed Treatment Short Course (DOTS) therapy as per WHO recommendations, monitored by the National Tuberculosis Elimination Program (NTEP). Follow-up extended through 6 months of treatment and 1-year post-treatment completion. Treatment outcomes were defined as favorable or unfavorable. Favorable treatment outcome (cure) was defined as negative results of sputum cultures at months 5 and 6 of treatment without recurrent disease during follow-up. Unfavorable treatment outcomes included treatment failure defined as positive sputum culture results at month 5 or 6, all-cause mortality, or recurrent TB within 12 months after initial cure. These participants did not receive any treatment for anemia.

### Multiplex assays

Circulating plasma cytokines and pro-fibrotic levels were measured in a subset of anemic (*n* = 288) and non-anemic (*n* = 195) TB individuals using multiplex Luminex assay (Bio-Rad Laboratories, Inc.). The analytes measured included cytokines [Interferon (IFN)-γ, Interleukin (IL)-2, Tumor Necrosis Factor (TNF-α), IL-4, IL-5, IL-6, IL-13, IFN-α, and IFN-β] and pro-fibrotic factors (Vascular endothelial growth factor (VEGF), Epidermal growth factor (EGF), Fibroblast growth factor (FGF-2), and Platelet-derived growth factor (PDGF)-AB/BB). The experiment was conducted according to the manufacturer's instructions (R&D Systems).

### Statistical analysis

Before analysis, the data was thoroughly checked for completeness and consistency. Continuous variables were examined for normality using the Shapiro-Wilks test and were found not to be normal. The data was then presented using frequency, percentages, median and quartiles. Measurements of central tendency utilized geometric means (GMs). Differences in continuous variables between the two groups were examined using the Wilcoxon rank sum test, while the relationship between groups and factors such as sputum smear grade, bilateral lung lesion, cavitary lesion, and TB treatment failure and relapse were examined using the Pearson chi-square test. Statistically significant differences between two groups were analyzed using the non-parametric Mann-Whitney *U*-test with Holm's correction for multiple comparisons. Generalized linear models with binomial regression and log-link functions were used to identify key factors. The selection of covariates for the regression model was determined based on data availability, a review of relevant literature, and the opinions of subject matter experts. Prevalence ratios (PR) and adjusted prevalence ratios (aPR) were calculated along with the corresponding 95% confidence intervals (CIs). Covariates with significant PR, were considered when adjusting for aPR. Data analysis was performed using STATA software, version 15.0 (StataCorp., Texas, USA), with all *P*-values considered two-sided and statistical significance set at the 0.05 α level.

## Results

### Study population characteristics

The study comprised 483 culture-confirmed TB individuals, with 288 positives (101 male, 187 female) for anemia and 195 negatives (90 male, 105 female). Median age was 45 years [interquartile range (IQR), 36.0–53.0] for participants with anemia and 47 years (IQR, 36.3–52.0) for participants without anemia. There were no statistically significant differences in age, BMI, smoking, alcohol use, and HbA1c between the TB subjects with anemia and those without anemia. However, significant differences were observed in gender (*p* = 0.0175; Table 1) and notable differences in certain hematological and biochemical parameters (Table 2). Individuals with anemia exhibited significantly lower levels of red blood cells (RBC; GM of 4.4 g/dL vs. 5.1 mg/dL; *p* < 0.0001), hemoglobin (Hb; GM of 11 vs. 14.3 g/dL; *p* < 0.0001), and hematocrit (HCT; GM of 34.9 vs. 42.4%; *p* < 0.0001), and elevated monocyte counts (GM of 708.2 vs. 620.1 cells/μL; *p* = 0.0404) compared to subjects without anemia. Additionally, biochemical parameters such as triglycerides (GM of 97.1 vs. 104.1 mg/dL; *p* = 0.0310), total cholesterol (GM of 157.3 vs. 168.2 mg/dL; *p* = 0.0083), LDL (GM of 89.6 vs. 95.4 mg/dL; *p* < 0.0001), total protein (GM of 7.9 vs. 8.2 g/dL; *p* = 0.0048), serum albumin (GM of 3.8 vs. 4.2 g/dL; *p* < 0.0001), and Vitamin D (GM of 15.4 vs. 17.4 IU; *p* = 0.0269) were significantly lower in individuals with anemia compared to subjects without anemia.

**Table 1:** Demographics and clinical characteristics of the study population.

**Variable**	**Overall, *N* = 483^a^**	**Participants with anemia, *N* = 288 (59.6%)^a^**	**Participants without anemia, *N* = 195 (40.4%)^a^**	***p*-value**
**Age in years, median (IQR)**	45.0 (36.0 – 52.0)	45.0 (36.0 – 53.0)	47.0 (36.3 – 52.0)	0.649^b^
**Age classification (in years)**, ***n*** **(%)**				0.787^c^
Up to 35 years	112 (23.2)	59 (24.1)	53 (22.3)	
36–45 years	136 (28.2)	64 (26.1)	72 (30.3)	
46–55 years	153 (31.7)	80 (32.7)	73 (30.7)	
>55 years	82 (17.0)	42 (17.1)	40 (16.8)	
**Gender**, ***n*** **(%)**				0.0175^c^
Female	292 (60.5)	187 (64.9)	105 (53.8)	
Male	191 (39.5)	101 (35.1)	90 (46.2)	
	20.0 (17.5 – 23.3)	20.3 (17.9 – 23.4)	19.9 (17.5 – 23.0)	0.356^b^
**Body mass index (kg/m**^2^**), median (IQR), and body mass index classification (kg/m**^2^**)**, ***n*** **(%)**				
Normal (18.5–24.9 kg/m^2^)	190 (39.3)	93 (38.0)	97 (40.8)	0.730^c^
Undernourished (< 18.5 kg/m^2^)	154 (31.9)	76 (31.0)	78 (32.8)	
Overweight (25.0–29.9 kg/m^2^)	91 (18.8)	49 (20.0)	42 (17.6)	
Obesity (≥30 kg/m^2^)	48 (9.9)	27 (11.0)	21 (8.8)	
	6.8 (5.7– 10.3)	6.7 (5.7 −10.4)	7.9 (5.8 – 10.4)	0.5014^b^
**Glycated hemoglobin (HbA1c) %, median (IQR), and diabetes mellitus (DM) (HbA1c%)**, ***n*** **(%)**				
No, DM (< 5.7%)	94 (19.5)	54 (22.0)	40 (16.8)	0.068^c^
Pre, DM (5.7–6.4%)	127 (26.3)	54 (22.0)	73 (30.7)	
DM (≥6.5%)	262 (54.2)	137 (55.9)	125 (52.5)	
**Smoking status**, ***n*** **(%)**				0.191^c^
Non-smoker	139 (28.8)	66 (26.9)	73 (30.7)	
Smoker	125 (25.9)	58 (23.7)	67 (28.2)	
Unknown	219 (45.3)	121 (49.4)	98 (41.2)	
**Alcohol use**, ***n*** **(%)**				0.464^c^
Yes	249 (51.6)	122 (49.8)	127 (53.4)	
No	75 (15.5)	36 (14.7)	39 (16.4)	
Unknown	159 (32.9)	87 (35.5)	72 (30.3)	
**Cavitary lung lesions**, ***n*** **(%)**				< 0.001^c^
No, cavitary lung lesions	198 (41.0)	123 (50.2)	75 (31.5)	
Cavitary lung lesions	285 (59.0)	122 (49.8)	163 (68.5)	
**Bilateral lung lesions**, ***n*** **(%)**				0.004^c^
No, bilateral lung lesions	96 (19.9)	36 (14.7)	60 (25.2)	
Bilateral lung lesions	387 (80.1)	209 (85.3)	178 (74.8)	
**AFB smear testing**, ***n*** **(%)**				< 0.001^c^
Smear –ve	63 (13.0)	47 (19.2)	16 (6.7)	
Smear +ve	420 (87.0)	198 (80.8)	222 (93.3)	
**TB treatment outcome**, ***n*** **(%)**				0.538^c^
Favorable outcome	448 (92.8)	229 (93.5)	219 (92.0)	
Treatment failure/relapse	35 (7.2)	16 (6.5)	19 (8.0)	

^a^Median (IQR) or frequency (%).

^b^Mann-Whitney test.

^c^Wilcoxon rank sum test; Pearson's Chi-squared test.

Anemia was defined as < 12 g/dL for Female and < 13 g/dL for Male.

**Table 2:** Hematological and biochemical parameters of the study population.

**Parameters**	**Participants with anemia (*n* = 288) GM (range)**	**Participants without anemia (*n* = 195) GM (range)**	***p*-value^a^**
WBC count, x10^3^cells/ul	97.4 (40–269)	94.1 (40–2000)	0.2656
Lymphocyte count, x10^6^cells/ul	1,889 (500–4,015)	2062.3 (560–5,445)	0.0533
Neutrophil count, cells/ul	6,077 (1,632–16,020)	6512.8 (2,646–14,000)	0.5622
Monocyte count, cells/ul	708.2 (146–2,421)	620.1 (182–1,870)	0.0404
RBC, g/dL	4.4 (3–6.4)	5.1 (4–6.7)	< 0.0001
Hb, g/dL	11 (6.3–12.9)	14.3 (13–20.1)	< 0.0001
Hematocrit, %	34.9 (22–57)	42.4 (26–58)	< 0.0001
Platelets, 10^3^/uL	344.2 (90–800)	323.1 (123–817)	0.0719
FBG, mg/Dl	129.2 (74–516)	140.5 (62–417)	0.0560
HbA1C, %	6.7 (4.5–15.3)	7.9 (4.9–17.7)	0.5014
Triglycerides, mg/dL	97.1 (50–275)	104.1 (42–348)	0.0310
Total cholesterol, mg/dL	157.3 (80–294)	168.2 (91–330)	0.0083
HDL, mg/dL	35.5 (17–69)	37.5 (21–66)	0.7647
LDL, mg/dL	89.6 (33–187)	95.4 (35–223)	0.0411
VLDL, mg/dL	31.1 (13–157)	32.9 (10–166)	0.0796
Urea, mg/dL	16.6 (5–79)	17.5 (7–57)	0.0506
Creatinine, mg/dL	0.8 (0.5–1.7)	0.9 (0.5–2.1)	0.0540
Total bilirubin, mg/dL	0.5 (0.3–2)	0.6 (0.3–2.1)	0.0648
Total protein, g/dL	7.9 (5.7–10.4)	8.2 (6.1–10.1)	0.0048
Serum albumin, g/dL	3.8 (2.3–5.4)	4.2 (2.5–5.2)	< 0.0001
Serum globulin, g/dL	4.0 (2.3–7)	3.9 (2.6–5.9)	0.0652
SGOT, U/L	18.0 (6–91)	17.9 (6–145)	0.9843
SGPT, U/L	14.6 (4–141)	15.6 (5–76)	0.0512
Alkaline phosphatase, U/L	269.9 (102–957)	259.4 (94–707)	0.9963
Vitamin D, IU	15.4 (3–47)	17.4 (3–67)	0.0269

^a^Mann-Whitney test.

GM, geometric mean; WBC, Whole blood cells; RBC, Red blood cells; Hb, Hemoglobin; FBG, Fasting blood glucose; HbA1c, Glycated hemoglobin; HDL, High density lipoprotein; LDL, Low density lipoprotein; VLDL, Very low-density lipoprotein; SGOT, serum glutamic-oxaloacetic transaminase; SGPT, Serum Glutamate Pyruvate Transaminase.

### Association of clinical co-morbidities with anemia in TB individuals

No significant differences were observed in age, BMI, smoking, alcoholism, or HbA1c) between the two groups (Table 3). However, significant differences were noted in gender (female). The PR for female individuals with anemia was 3.21 (95% CI: 1.52–3.31; *p* = 0.009), and this association remained significant after adjusting for possible confounders (aPR 2.50, 95% CI: 1.90–2.40; *p* = 0.028).

**Table 3:** Association of clinical co-morbidities with anemia in TB individuals.

**Variable**	**Anemia/TB PR (95% Cl)**	***p*-value**	**Anemia/TB aPR (95% Cl)**	***p*-value**
**Socio-demographic characteristics-Sex**				
Male	Reference	1.009	Reference	0.028
Female	3.21 (1.52–3.31)		2.50 (1.90-2.40)	
**Age, years**				
18–34	Reference	0.879	Reference	0.981
35–44	1.61 (0.81–1.96)		1.60 (0.92–2.03)	
45–54	1.40 (0.76–2.21)		1.76 (0.87–2.01)	
≥55	1.10 (0.61–2.02)		1.11 (0.46–1.98)	
**Smoking**				
No	Reference	0.991	Reference	0.541
Yes	2.01 (0.89–3.25)		1.20 (0.76–2.54)	
Unknown	1.98 (1.74–2.87)		1.91 (0.82–3.10)	
**Alcoholism**				
No	Reference	0.928	Reference	0.571
Yes	2.50 (0.71–4.20)		1.80 (0.40–3.71)	
Unknown	2.81 (0.41–3.50)		1.30 (0.51–2.60)	
**BMI (kg/m** ^2^ **)**				
Normal (18.5–24.9)	Reference	0.0781	Reference	0.211
Under nutrition (< 18.5)	3.20 (0.51–2.80)		2.54 (0.61–1.71)	
Overweight (25.0–29.9)	2.68 (0.31–2.41)		1.52 (0.87–2.50)	
Obesity (≥30.0)	1.60 (0.86–2.71)		1.7 (0.71–4.51)	
**HbA1c (%)**				
NDM (< 5.7)	Reference	0.741	Reference	0.438
PDM (>5.7– < 6.4)	1.52 (0.75–2.01)		1.92 (0.73–0.90)	
DM (>6.4)	1.10 (0.61–1.47)		1.30 (0.61–0.10)	

PR, prevalence ratio; aPR, adjusted prevalence ratio; CI, Confidence interval; BMI, Body mass index; HbA1c, Glycated hemoglobin; NDM, Non-diabetes mellitus; PDM, Pre-diabetes mellitus; DM, Diabetes mellitus.

### Anemia is associated with increased radiographic TB disease severity and greater bacterial burdens

Anemia was significantly associated with an increased risk of cavitary disease (PR, 4.62; 95% CI, 3.04–7.08; *p* < 0.001) but not of bilateral lung lesions (PR, 2.21; 95% CI, 0.98–3.12; *p* = 0.287). After adjusting for confounding variables, anemia remained significantly associated with a higher risk of cavitation (aPR, 3.36; 95% CI, 1.95–5.68; *p* < 0.001), indicating increased TB disease severity in individuals with anemia. Additionally, anemia was significantly associated with an elevated risk of higher smear grades (PR, 5.51; 95% CI, 3.45–9.34; *p* < 0.001). This association persisted after adjusting for confounders, with anemia remaining significantly associated with increased smear grades (aPR, 4.01; 95% CI, 2.22–6.63; *p* < 0.001), indicating higher bacterial burdens in TB patients with anemia (Table 4).

**Table 4:** Association of anemia with bacterial burden, disease severity, and treatment failure/relapse in TB.

**Outcome variable**	**Anemia/TB PR (95% Cl)**	***p*-value**	**Anemia/TB aPR (95% Cl)**	***p*-value**
Sputum smear grade	5.51 [3.45–9.34]	< 0.001	4.01 [2.22–6.63]	< 0.001
Bilateral lung lesions	2.21 [0.98–3.12]	0.287	1.96 [0.84–4.52]	0.354
Cavitary lung lesions	4.62 [3.04–7.08]	< 0.001	3.36 [1.95–5.68]	< 0.001
TB treatment failure/relapse	1.72 [1.11–2.61]	0.019	1.61 [1.31–2.19]	0.046

TB, tuberculosis; PR, prevalence ratio; aPR, adjusted prevalence ratio; CI, confidence interval.

### Anemia is associated with increased risk of unfavorable TB treatment outcomes

Anemia was significantly associated with an increased risk of unfavorable treatment outcomes (PR, 1.72; 95% CI, 1.11–2.61; *p* = 0.019). This association persisted even after adjusting for confounding variables, with anemia remaining significantly associated with unfavorable treatment outcomes (aPR, 1.61; 95% CI, 1.31–2.19; *p* = 0.046). These findings indicate a heightened risk of treatment failure or TB recurrence in TB patients with anemia (Table 4).

### Anemia is associated with altered levels of cytokines and pro-fibrotic factors in TB

**Figure 1:**
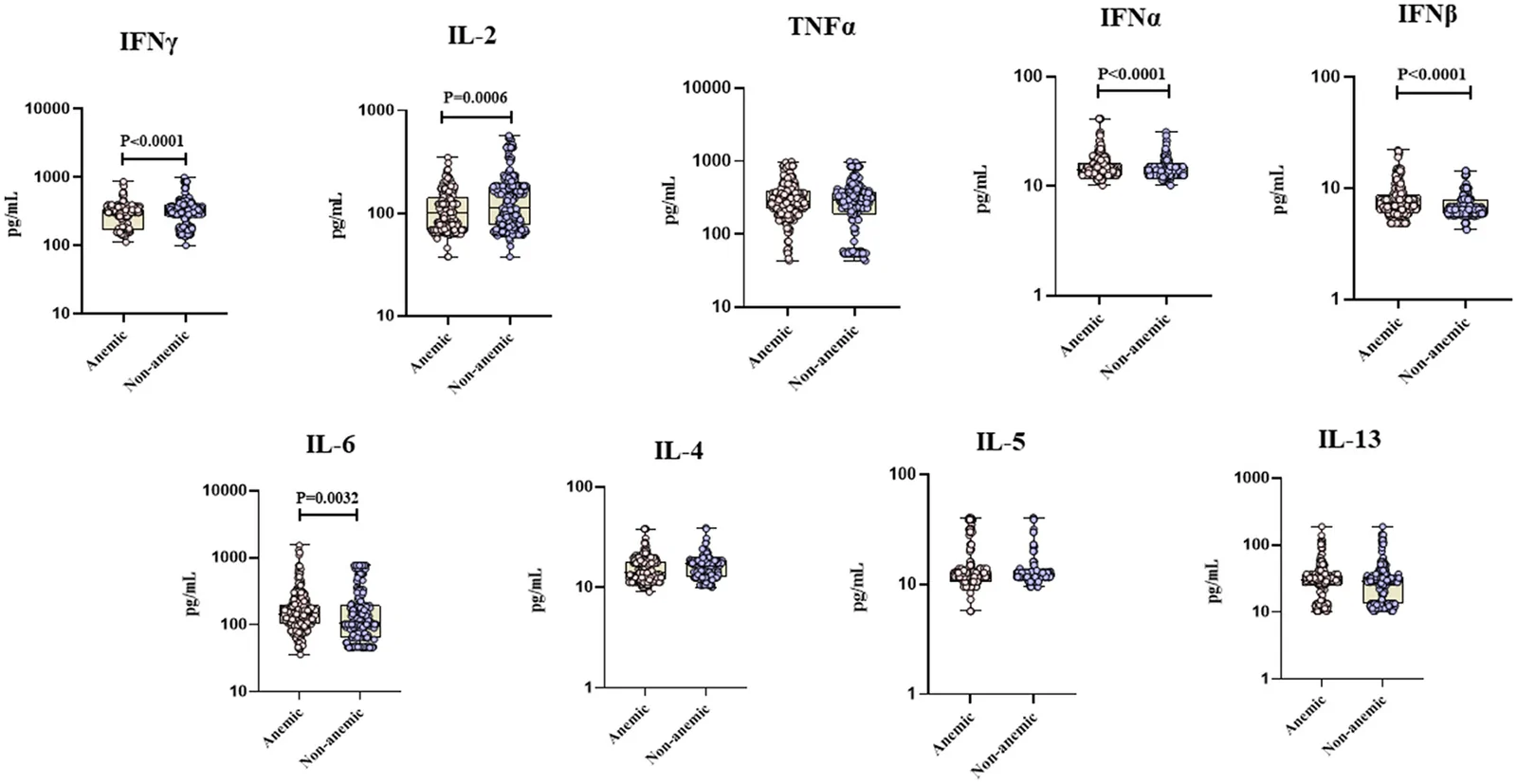
Anemia is associated with altered levels of cytokines in TB individuals. The figure illustrates the cytokine profile in anemic and non-anemic TB individuals. Circulating plasma cytokines including Interferon gamma (IFN γ), Interleukin-2 (IL-2), Tumor necrosis factor alpha (TNF-α), Interferon alpha (IFN α), Interferon beta (IFN β), Interleukin-6 (IL-6), Interleukin-4 (IL-4), Interleukin-5 (IL-5), and Interleukin-13 (IL-13) were measured. Each data point represents an individual subject, with the bar indicating the geometric mean (GM) cytokine level. Statistical analysis was performed using the Mann–Whitney *U*-test.

**Figure 2:**
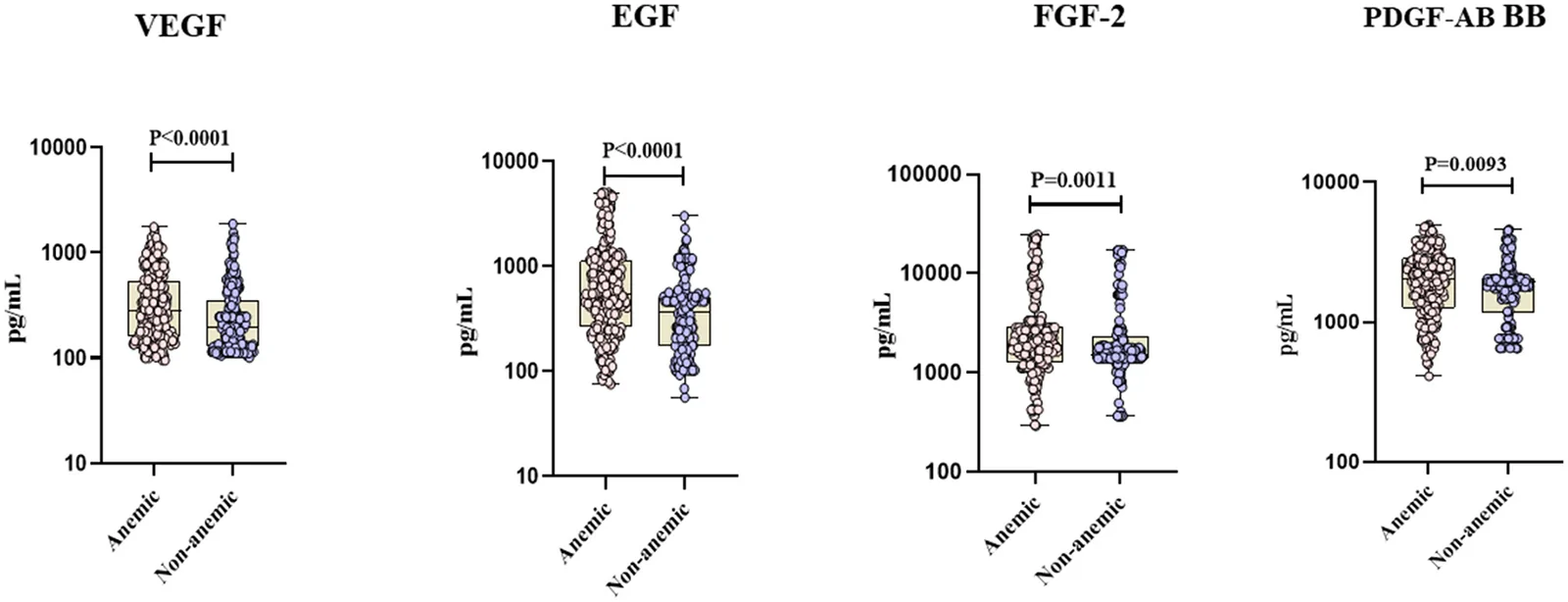
Anemia is associated with heightened levels of pro-fibrotic factors in TB individuals. The figure illustrates the pro-fibrotic factors profile in anemic and non-anemic TB individuals. Circulating plasma pro-fibrotic factors including Vascular endothelial growth factor (VEGF), Epidermal growth factor (EGF), Fibroblast growth factor 2 (FGF-2), and Platelet-derived growth factor (PDGF-AB BB) were measured. Each data point represents an individual subject, with the bar indicating the geometric mean (GM) cytokine level. Statistical analysis was performed using the Mann–Whitney *U*-test.

The circulating levels of TNF-α, IL-4, IL-5, and IL-13 did not significantly differ between the two groups. However, pro-inflammatory cytokines [IFN-α (GM of 15.18 vs. 13.91 pg/ml, *p* < 0.0001), IFN-β (GM of 6.69 vs. 6.53 pg/ml, *p* < 0.0001), IL-6 (GM of 146.53 vs. 133.69 pg/ml, *p* = 0.0032)], and pro-fibrotic factors [VEGF (GM of 217 vs. 154.71 pg/ml, *p* < 0.0001), EGF (GM of 384.22 vs. 317.56 pg/ml, *p* < 0.0001), FGF-2 (GM of 2,584.82 vs. 2,000.96 pg/ml, *p* = 0.0011), and PDGF-AB/BB (GM of 1,758.71 vs. 1,601.31 pg/ml, *p* = 0.0093)] were significantly elevated in TB individuals with anemia compared to those without anemia. Conversely, the circulating plasma levels of type 1 cytokines [IFN-γ (GM of 275.56 vs. 313.81 pg/ml, *p* < 0.0001), IL-2 (GM of 104.75 vs. 121.96 pg/ml, *p* = 0.0006)] were significantly diminished in TB individuals with anemia compared to those without anemia (Figures 1, 2). Thus, anemia is associated with heightened levels of pro-inflammatory cytokines and pro-fibrotic factors and diminished levels of type 1 cytokines in TB individuals.

## Discussion

To enhance TB management, targeted interventions must investigate the risk factors associated with disease progression and poor treatment outcomes (30). Anemia is a prominent comorbidity with TB (19). However, existing literature on the relationship between anemia and disease severity is scarce and inconsistent. While some studies suggest that anemia does not significantly predict TB risk (31–33), others identify it as a potential risk factor (34–36). This discrepancy underscores the necessity for robust, well-designed studies with larger sample sizes and standardized methodologies.

Consistent with prior research, our study revealed a substantial burden of anemia among TB patients in our cohort (37, 38). Notably, we observed higher rates of anemia among female TB patients compared to males, likely attributed to physiological differences, dietary habits, and variation in health-seeking behavior between genders (39). In contrast to non-anemic subjects, individuals with anemia exhibited a marked increase in monocyte levels and significant decreases in Hb, HCT, and RBC. Recent studies have linked elevated monocytes to poor prognosis and delayed pulmonary cavity closure in TB patients with anemia (10). Experimental evidence suggests that reduced Hb levels in anemic TB patients may result from the severity of TB infection and inflammation, impacting erythropoiesis and exacerbated by iron deficiency (40, 41). Hence, individuals with TB-related anemia may have a longer time for the proliferation and accumulation of Mtb, exposing them to inflammation for a longer time (12). The decreased production of RBC might result in reduced oxygen-carrying capacity and tissue hypoxia, which may have an impact on cytokine levels, leukocyte function, bone marrow function, and tissue destruction in TB (42, 43). Furthermore, our findings revealed that subjects with anemia had significantly lower levels of vitamin D and serum albumin compared to non-anemic subjects. Low serum albumin levels serve as a predictor of anemia and indicate the severity of inflammation (44, 45). The biological plausibility of lower vitamin D in anemia is supported by evidence suggesting that vitamin D regulates hepcidin production, thereby controlling iron homeostasis and erythropoiesis (46, 47).

Biomarkers for TB unfavorable treatment outcomes can play a major role in identifying novel TB intervention strategies (48–54). Cytokines are critical in the host defense against mycobacterial infections, serving as markers of disease severity and bacterial burden in active TB (55–57). Research shows that LBMI significantly impacts both acquired and innate host defense mechanisms, increasing susceptibility to TB (58–62). Our findings add to this knowledge by demonstrating that TB with coexistent anemia is associated with reduced levels of type 1 cytokines and increased pro-inflammatory and pro-fibrotic factors, potentially heightening TB risk. Our data indicate that TB patients with anemia have lower circulating levels of type 1 cytokines (IFNγ and IL-2), suggesting impaired protective immunity (63, 64). The reduced production of these cytokines in anemic individuals suggests a higher risk for severe TB due to weakened cell-mediated immunity, aligning with studies reporting lower type 1 cytokine levels in individuals with LBMI and TB compared to those with normal or high BMI (58, 62).

Loss of immune control in TB often results from excessive pro-inflammatory cytokine production, leading to neutrophil infiltration and pathological inflammation. This promotes granuloma remodeling and lung tissue destruction (65). We found significantly elevated pro-inflammatory cytokines (IL-6, IFNα, and IFNβ) in TB patients with anemia compared to non-anemic individuals. This aligns with previous research linking high IL-6 levels to inflammation-related anemia, which inhibits iron absorption and exacerbates TB progression (66, 67). Additionally, high IL-6 concentrations are associated with anemia in TB/HIV co-infected patients (68). Pro-fibrotic factors are crucial in bacterial infection processes. Our study showed increased levels of pro-fibrotic factors (VEGF, EGF, FGF-2, and PDGF-AB BB) in anemic individuals compared to non-anemic individuals. VEGF, associated with pleural inflammation and fibrosis in TB patients, has been found at elevated levels in smear-positive and culture-positive TB subjects (69). Systemic VEGF levels also rise significantly in TB patients with cavitations and bilateral disease involvement (70).

In this study, rigorous control was exercised over several factors known to influence disease severity and bacterial burdens, such as age, BMI, diabetes, smoking status, and alcohol use. The findings of this study provide valuable insights into the association between anemia and TB disease severity. Notably, our study revealed several key findings that warrant further exploration. We observed that TB patients with coexistent anemia exhibit more severe disease manifestations, including lung cavitation, indicative of advanced TB disease. These findings align with previous research suggesting that such lesions negatively impact patients and may lead to poor treatment outcomes, relapses, and drug resistance (71). Our results revealed a strong correlation between anemia and elevated bacterial burdens in TB patients, a key indicator of transmission (10). Our data further confirm that TB individuals with anemia were at a significantly higher risk of experiencing unfavorable treatment outcomes, including treatment failure or TB recurrence. This finding aligns with previous research indicating that anemic patients with TB-HIV co-infection exhibit poor treatment outcomes and a heightened degree of inflammatory perturbation (72).

Our study suffers from the limitation of not measuring red cell indices (MCV, MCH, and MCHC) or biochemical measures (iron, ferritin, hepcidin, and transferrin) to assess the type of anemia. Another limitation of our study is that cytokine levels exhibit a great degree of overlap between groups and that there is variability in the responses of different individuals in the same group. It is theoretically possible that other factors not examined in this study could have contributed to the differential responses. Nevertheless, our study offers novel insights into the immunological underpinnings of the anemia-TB comorbidity.

## Conclusion

Our study reveals intricate interactions between anemia and disease severity, bacterial burdens, and treatment outcomes in TB patients. Importantly, our data highlights the significant association of anemia with the cytokine milieu in TB, suggesting a plausible biological mechanism for the increased disease severity observed in TB individuals with coexistent anemia. Our findings highlight the critical need for further research and interventions aimed at addressing the complex interplay between anemia and TB to optimize patient outcomes and advance TB control efforts.

## Data Availability

The raw data supporting the conclusions of this article will be made available by the authors, without undue reservation.
